# The Behavioral and Clinical Effects of Therapeutic Lifestyle Change on Middle-aged Adults

**Published:** 2005-12-15

**Authors:** Steven G Aldana, Roger L Greenlaw, Audrey Salberg, Hans A Diehl, Ray M Merrill, Camille Thomas, Seiga Ohmine

**Affiliations:** College of Health and Human Performance, Brigham Young University; SwedishAmerican Center for Complementary Medicine, Rockford, Ill; SwedishAmerican Center for Complementary Medicine, Rockford, Ill; Lifestyle Medicine Institute, Loma Linda, Calif; SwedishAmerican Center for Complementary Medicine, Rockford, Ill; College of Health and Human Performance, Brigham Young University, Provo, Utah; Department of Molecular and Microbiology, Brigham Young University, Provo, Utah

## Abstract

**Introduction:**

Chronic diseases such as cancer, cardiovascular disease, stroke, and diabetes are responsible for most deaths in the United States. Lifestyle factors — poor nutrition, sedentary living, and tobacco use — appear to play a prominent role in the development of many chronic diseases. This study determined the behavioral and clinical impact of a therapeutic lifestyle-modification intervention on a group of community volunteers.

**Methods:**

Participants included 348 volunteers aged 24 to 81 years from the Rockford, Ill, metropolitan area who participated in a randomized clinical trial. The intervention group attended a 40-hour educational course delivered as lectures during a 4-week period. Participants learned the importance of making better lifestyle choices and how to make improvements in nutrition and physical activity. Changes in nutrition, physical activity behavior, and several chronic disease risk factors were assessed at baseline and 6 months.

**Results:**

Intervention participants showed significant 6-month improvement in all nutrition and physical activity measures except calories from protein and whole-grain servings and all clinical measures except blood glucose, total cholesterol, triglycerides, and high-sensitivity C-reactive protein. Total cholesterol and low-density lipoprotein cholesterol were worse after 6 months in both groups but only significantly worse in the control group. The control group experienced small but significant improvements in systolic and diastolic blood pressure and high-density lipoproteins. Change-score comparisons between the intervention and control groups were significant for all nutrition and physical activity variables except total steps per week and daily sodium intake and were also significant for the clinical measures of weight, body fat, and body mass index.

**Conclusion:**

This therapeutic lifestyle-modification program can significantly improve nutrition and physical activity behavior and can reduce many of the risk factors associated with common chronic diseases.

## Introduction

Chronic diseases such as cancer, cardiovascular disease, stroke, and diabetes are responsible for most deaths in the United States (1). Between 70% and 90% of these deaths are believed to be caused by poor nutrition, sedentary living, and tobacco use and are preventable (2-4). These lifestyle factors appear to play a prominent role in the mechanisms and processes that lead to the development of many chronic diseases. The largest reductions in chronic disease prevalence in the United States will be achieved when individuals adopt and maintain lifestyles that include a healthy diet and regular physical activity.

During the 1980s, Nathan Pritikin conducted several in-patient lifestyle-change programs that documented how a low-fat, high–complex-carbohydrate, high-fiber diet and regular exercise could improve blood lipid levels and insulin sensitivity (5-7). Variations of this holistic approach to preventing and arresting chronic diseases have more recently been evaluated in randomized clinical trials such as the PREMIER clinical trial (8), the DASH dietary study (9), and other trials in the United States, United Kingdom, and New Zealand (10-12). Most of these trials used inpatient treatment or controlled feeding to encourage and monitor changes in diet and physical activity. All of them demonstrated reductions in cardiovascular risk factors, including obesity, blood pressure, and blood lipid levels.

The Coronary Health Improvement Project (CHIP) was created with the goal of reducing chronic diseases and improving the overall health of the public by providing a lifestyle-change program to both the community and the workplace (13). The CHIP is a 40-hour live-lecture educational course that highlights the importance of making better lifestyle choices for reducing chronic disease risk factors. A one-group pretest–posttest analysis of the program revealed that after 4 weeks, participants significantly reduced their blood pressure, blood glucose, body weight, and total and low-density lipoprotein (LDL) cholesterol (13). This exploratory study demonstrated that the program had the potential to improve not only coronary risk factors but also the risks associated with cancer, diabetes, and the metabolic syndrome. These results were repeated in a quasi-experimental design that included results from six groups of working adults (14).

A large randomized clinical trial was initiated to further explore the effect of the CHIP (15). Six-week results from this study revealed that adults who completed the program improved their nutrition and physical activity behavior and reduced cardiovascular disease risk factors (15). We present the behavioral and clinical changes that participants in this therapeutic lifestyle-change program experienced after 6 months.

## Methods

### Subject recruitment and study design

**Figure 1:**
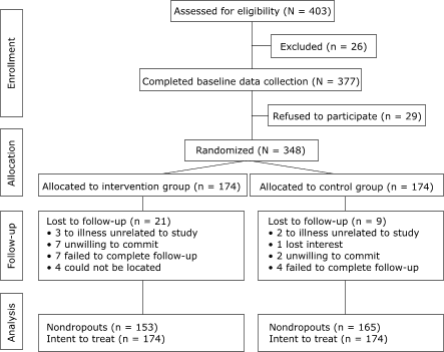
Process for a therapeutic lifestyle-modification intervention with a group of community volunteers, Rockford, Ill.

Recruitment was conducted by the SwedishAmerican Center for Complementary Medicine (SACCM) using targeted advertising, marketing through the SwedishAmerican Health System Centers of Excellence, CHIP alumni groups, corporate client sites, and the SwedishAmerican Health System. Recruitment efforts were aimed at adults (aged at least 18 years) in the greater Rockford, Ill, metropolitan area. To be enrolled in the study, each participant had to be willing to start participating in the program in 1 month or in 7 months. Figure 1 shows participant progress through the study. Eligible and interested participants provided informed consent. Participants were highly encouraged to participate with a spouse or significant other and were randomized as a paired unit. All other participants were randomized as individual units. The allocation sequence was created using a random number generator. Program sign-up, randomization, and group assignments were made by the study coordinator. The study was approved by the Institutional Review Board of the SwedishAmerican Health System on August 29, 2002.

### Intervention

The intervention for this study was a live version of the CHIP (13). Participants met for 4 weeks — 4 times each week for 2 hours — to receive instruction during April 2003. The curriculum included the following topics: modern medicine and health myths, atherosclerosis, coronary risk factors, obesity, dietary fiber, dietary fat, diabetes, hypertension, cholesterol, exercise, osteoporosis, cancer, lifestyle and health, the Optimal Diet, behavioral change, and self-worth.

In conjunction with CHIP lectures, participants received a textbook and workbook that closely followed the curriculum topics and included assignments with learning objectives for every topic. Copies of these materials can be obtained from CHIP at www.chipusa.org. Assignments were designed to help participants understand and integrate the information presented. Dietitians and medical professionals spoke to the group weekly, introducing them to the latest nutritional and medical information related to the prevention of chronic diseases. Participants had access to scheduled shopping tours and cooking demonstrations given by a dietitian.

The diet guidelines approximate the recommendations previously used in the Pritikin program (5-7), and the exercise guidelines are from the Surgeon General's Report on Physical Activity and Health (16). Participants were encouraged to follow preset dietary and exercise goals. The dietary goal was to adopt a more plant-food–based diet that emphasizes as-grown, unrefined food. Participants were encouraged to eat the following foods: whole grains, legumes, vegetables, and fresh fruits. In addition, the diet was low in fat (less than 20% of energy), animal protein, sugar, and salt; very low in cholesterol; and high in fiber. Concurrently, program participants were encouraged to work toward walking or exercising for at least 30 minutes each day. Participants were given a pedometer and encouraged to keep an exercise log to record the miles walked each day. In addition, at the completion of the program, participants were encouraged to join the Rockford CHIP alumni association for an annual cost of $25 for an individual or $35 for a couple. The purpose of the alumni organization was to help prevent relapse and help participants maintain their new behaviors. Alumni receive a monthly newsletter that contains news of health-promoting community events such as healthy dinners, walking groups, and support-group meetings. The alumni were encouraged to attend special lectures on healthy living and ways to avoid relapse.  

The primary objectives of this therapeutic lifestyle-change program were to improve cognitive understanding of the importance of healthy lifestyles, nutrition, and physical activity behavior and reduce risk factors associated with hypertension and cardiovascular disease. The cost to participate in the entire CHIP was $395 per person or $595 per couple.

### Measures

Variables gathered included cognitive and behavioral measurements and physiologic outcomes related to chronic disease. Demographic data were collected at baseline in April 2003. Attendance at each of the classes was tracked and averaged. Participants attended an average of 89% of the classes.

The intervention was designed to assist individuals in adopting healthy eating and physical activity behaviors. To assess dietary intake, the Block 98 full-length dietary questionnaire was used (Block 98.2, Block Dietary Data Systems, Berkeley, Calif). The Block 98 questionnaire has been extensively studied and validated (17). The questionnaire contains self-reported data and is optically scanned and scored. The questionnaire measures the following variables (in addition to others) on a per-day basis: nutrients obtained from food; percentage of calories from fats, carbohydrates, and protein; fiber from different sources; and food group servings per day.

To ascertain energy expenditure contributed by physical activity, a 7-day self-recorded pedometer log was maintained by each participant. Participants wore the Walk4Life Model 2000 Life Stepper pedometer (Walk4Life Inc, Plainfield, Ill) on a belt at the right hip directly above the right kneecap each day for 7 days. Immediately before going to bed, participants recorded the number of steps for the day and reset the pedometer. Strike counts from pedometers are a valid and reliable method of monitoring and measuring free-living physical activity (18).

The primary outcome variables for this study included several chronic disease risk factors. The following data were collected from April to October 2003. Blood was drawn from participants (after a 12-hour fast) by phlebotomists from the SwedishAmerican Health System's outpatient laboratory using a vacutainer (Becton-Dickinson Vacutainer Systems, Rutherford, NJ). Samples were allowed to clot and were centrifuged. Clinical analyses were completed at the SwedishAmerican Health System laboratory. Lipid analysis followed the lipid standards provided by the Centers for Disease Control and Prevention. Glucose, total cholesterol, high-density lipoprotein (HDL), and triglyceride concentrations were determined using Beckman-Coulter LX-20 instrumentation (Beckman Coulter, Inc, Fullerton, Calif). Glucose measurements were obtained with the oxygen-rate method using a Beckman oxygen electrode; cholesterol measurements were obtained with the timed-endpoint enzymatic method using cholesterol oxidase; triglyceride measurements were obtained with the timed-endpoint enzymatic method using glycerol kinase; and HDL measurements were obtained with the homogeneous timed-endpoint method using polyanion detergent to separate HDL and non-HDL lipids. For participants with triglyceride values below 400, LDL values were calculated as follows: LDL = total cholesterol – HDL – (triglycerides/5) (19). High-sensitivity C-reactive protein (CRP) measurements were determined using a microplate protocol based on a latex-bead–enhanced immunoturbidity assay (20). Glucose measurements were determined using a Kodak Ektachem (Kodak, Rochester, NY). Trained program staff took blood pressure measurements. Blood pressure was measured in participants after a 5-minute rest, using the guidelines set forth by the American Heart Association. Weight and height were measured using standard medical weight and height scales recently calibrated by the biometrics department of the SwedishAmerican Health System. Percentage of body fat was estimated with Tanita TBF-300A Body Composition Analyzer/Scale using bioelectrical impedance analysis (Tanita, Tokyo, Japan) (21). Body mass index (BMI) was determined using the following formula: weight (kg)/height (m^2^).

### Statistical analyses

Cross-tabulations were used to perform bivariate analyses between selected variables, with statistical significance based on the chi-square test for independence. For testing differences in means, *t* tests were used. Because multiple pair-wise tests were performed, an adjusted α was used to minimize the overall probability of committing a type I error. The modified α is .0001, based on the Bonferroni correction, 28 pair-wise tests, and α = .05. This conservative α was used to determine significance for data in Tables 2 through 7. Risk factor cut-points (Tables 6 and 7) were previously established (22,23) and categorized accordingly. Results are based on the intent-to-treat method in which all participants were retained in the analyses. Where participant data were lost to follow-up, the last-test carry-forward method was applied to the participant's most recent data. The results did not differ significantly when participants lost to follow-up were dropped from the analyses. These results are not reported. Analyses were performed using SAS 9.0 (SAS Institute Inc, Cary, NC). Procedure statements used in SAS for assessing the data were PROC UNIVARIATE, PROC FREQ, PROC TTEST, and PROC GLM.

## Results

There were 318 participants who completed both baseline and 6-month evaluations. An additional 30 completed the baseline evaluation but not the 6-month evaluation. Of these lost to follow-up, 21 were in the intervention group, and 9 were in the control group (Figure 1).

Analyses were based on 348 participants. Ages ranged from 24 to 81 years, with little difference in the mean age between intervention and control groups (50.1 years, intervention group; 50.8 years, control group, *t*_346_ = −0.57, *P* = .57). A description of participants in both intervention and control groups is presented according to selected demographic characteristics in Table 1. There were no statistically significant differences between groups for these variables. Within each group, the majority of participants had the following characteristics: female, white, married, an annual family income of at least $60,000, and at least some college education. Of the intervention participants, 47 (27%) joined the CHIP alumni association.

Because the unit of randomization was *pairs* for those who participated with a partner and *individuals* for those who participated as individuals, comparisons were made of the effect of the program between pairs and individuals. Of the 348 randomized participants, 146 (42%) participated as pairs. There were no significant differences in the outcomes of pairs and individuals. After 6 months, participants in the intervention group experienced significant improvements in all physical activity and nutrition variables except calories from protein and whole-grain servings (Table 2). Changes in the control group were generally not statistically significant, or they were much smaller in magnitude than the changes in the intervention group. For each variable except total steps per week and daily sodium intake, the change observed in physical activity or nutrition was significantly greater for participants in the intervention group compared with the control group (Table 3). The control group consumed significantly more fat calories and fewer whole-grain servings at 6-month follow-up compared with the control group at 6-month follow-up.

After 6 months, participants in the intervention group showed significant reductions in BMI, weight, body fat, systolic and diastolic blood pressure, and resting heart rate (Table 4). The control group experienced significant improvements in systolic and diastolic blood pressure and HDL, but total cholesterol and LDL were significantly worse. For BMI, weight, and body fat, changes were significantly greater for participants in the intervention group compared with the control group (Table 5).

Mean baseline, 6-month, and change in mean scores are presented according to standard health risk cut-points for the risk factor variables according to intervention group (Table 6) and control group (Table 7). This analysis stratifies results according to risk status. Individuals with low risk would not be expected to experience large changes, but risk values considered to be high would be expected to change significantly. For the intervention group, the distributions favorably changed between baseline and 6 months for BMI, systolic blood pressure, and diastolic blood pressure. Corresponding significant change in the distribution between baseline and 6 months was observed in the control group for systolic blood pressure and diastolic blood pressure but not for BMI. Favorable changes in risk behaviors were generally higher and more likely to be significant for individuals in the intervention group than for individuals in the control group.

Whereas total cholesterol significantly increased between baseline and 6 months for participants in the control group, no significant difference was observed in the intervention group. For both intervention and control groups, total cholesterol significantly increased among participants with total cholesterol in the normal range and decreased (but not significantly) for those with cholesterol in the high-risk category. Cholesterol medication played a minimal role in the change observed in cholesterol. At baseline, there were 77 participants in the intervention group who reported using blood pressure medication. At 6 months, 60 participants (75%) indicated no change in their medication over the study period, 9 participants (11.2%) indicated a dosage increase, and 11 participants (13.8%) indicated a dosage decrease. There was not a significant difference in the use of blood pressure medication from baseline to 6 months between the intervention and control groups (χ^2^_1_ = 1.14, *P* = .56).

## Discussion

Therapeutic lifestyle change can result in significant improvements in nutrition and physical activity behavior and reductions in many cardiovascular disease risk factors. Six months after the intervention began, program participants continued to demonstrate dramatic improvements in nutrition and physical activity behavior. Increases in the number of servings of fruit and vegetables and whole grains, increases in physical activity, and decreases in dietary sodium are likely responsible for the improvements in both systolic and diastolic blood pressure. Intervention group participants consumed 2.3 more servings of fruit and vegetables per day at 6 months compared with baseline. In the PREMIER study (8), participants who completed a behavior-change program and adopted the DASH diet increased fruit and vegetable servings by 3.0 servings after 6 months. Those PREMIER program participants decreased their percentage of calories from fat by 9.5% and lost an average of 5.8 kg of body weight. This compares to a percentage fat reduction of 8.2% and a 4.5 kg weight loss for intervention participants in the present study.

At baseline, the intervention group included 77 participants who were at least diastolic prehypertensive at 6 months; this number decreased by 44% to 43 participants at 6 months (Table 6). The number of intervention-group participants who were at least systolic prehypertensive at baseline declined by 20%, from 122 participants at baseline to 98 at 6 months. The average reductions in blood pressure were greater than the reductions reported in the DASH study (9) and comparable with the results of the PREMIER clinical trial (8).

Previous reports of the CHIP intervention showed sharp improvements in blood lipid levels at 6 weeks, but most of these changes disappeared at 6 months (7). Other therapeutic lifestyle trials that lasted longer than 3 months and included lipid outcomes reported similar findings (10-12,24). In this study, dietary cholesterol among the intervention group was reduced by 122 mg/day (a 56% reduction), and dietary saturated fat was cut by half. Despite these favorable changes in dietary cholesterol precursors, a return to previous lipid levels suggests that there is a significant increase in endogenous cholesterol, most of which appears to be LDL cholesterol (25). It is also possible that these changes in blood lipid levels were affected by seasonal variation. Without more accurate measures of endogenous cholesterol biosynthesis, it is impossible to determine the exact cause of the cholesterol increase (26).

Pedometer data show that program participants increased physical activity by 30%. The average number of steps for the intervention group after 6 months did not meet the recommended 10,000 steps per day (27). For this predominately middle-aged and obese population, however, an increase in physical activity of 30% likely contributed to risk factor reductions. When combined with diet changes, improvement in physical activity is the likely explanation for the percentage decreases in BMI (−5%), weight (−5%), and percentage body fat (−6%) among the intervention group. Improved physical activity was also associated with a significant decrease in resting heart rate, a correlated measure of cardiorespiratory fitness thought to be caused by increased heart size, blood volume, stroke volume, and cardiac output (28). 

Poor nutrition and sedentary living are associated with a constellation of risk factors, some identified in the metabolic syndrome, and all linked to common chronic diseases (29). Improvements in nutrition and physical activity are associated with significant reductions in diabetes risk as whole body glucose tolerance improves, insulin sensitivity increases, and the amount of glucose transporter (GLUT4) increases (30). The number of individuals with diabetes (glucose ≥126 mg/dL) in the intervention group was reduced by 19%, demonstrating that this therapeutic lifestyle-change program improves insulin sensitivity. Similar results were reported by other lifestyle trials reporting glucose findings (11,12). 

These improvements in behavior and risk are not unexpected because the intervention lectures were structured on the health belief and transtheoretical models. Video clips, testimonials, role playing, short presentations from physicians, social support strategies, food selection and planning activities, and other behavior-change–driven pedagogical activities helped to encourage participants to enthusiastically evaluate personal behaviors and commit to make lifestyle changes.

Most of the participants were white and sufficiently self-motivated to volunteer to participate in the intervention. On average, participants were slightly more educated than the community average. Participants had lifestyles that permitted them to attend most, if not all, of the classes. This is evident in the high rate of attendance to this time-intensive program. These delimitations threaten the generalizability of these findings and make application of the intervention to other populations problematic. Because the participants were self-selected, the results from this intervention may represent a best-case scenario.

Despite the apparent effect of this intervention, there are some shortcomings associated with the study design. Both the physical activity and nutrition data were self-reported. For some variables, the control group also experienced significant improvement. Significant decreases were observed in the control group in percentage of fat calories and dietary-fat grams, sodium grams, and total calories as well as small increases in total steps. In addition, the control group experienced similar improvement in blood pressure compared with the intervention group. There are more than 27 restaurants in the Rockford metropolitan area that offer healthy, CHIP-recommended menu items, which could have contributed to improvements in the control group. When conducting lifestyle trials, the question of what to do with the control group is difficult to answer because there is no such thing as a lifestyle placebo. After participants were assigned to an intervention or control group, some control-group participants expressed happiness with their assignment because they had personal or work-related conflicts that would have prohibited them from participating in the intervention group. Others were disappointed in their control-group assignment but realized when they agreed to participate in the research study that there was always the chance that they would have to wait to participate in the program.

This study indicates that an intervention that uses various behavior modification tools, such as live lectures, workbooks, and professional advice, and is implemented among a group of middle-aged volunteers can result in reduced risk factors for cardiovascular disease after 6 months. Further research is needed to examine the effects of the program on other populations.

## Figures and Tables

**Table 1:** Comparison of Demographic Characteristics of Intervention and Control Groups in a Therapeutic Lifestyle-Modification Program

**Characteristic**	**Interventionn = 174**		**Controln = 174**		**χ^2^ *_df_* (*P*)**

	**No.**	**%**	**No.**	**%**	

**Sex**					

Male	47	27.0	51	29.3	χ^2^_1_ = 0.2 (.63)
Female	127	73.0	123	70.7	

**Race**					

White	167	96.0	160	93.0	χ^2^_2_ = 2.9 (.23)
Black	4	2.3	10	5.8	
Other	3	1.7	2	1.2	

**Marital status**					

Never married	12	6.9	20	11.6	χ^2^_3_ = 3.0 (.39)
Married	138	79.8	127	73.4	
Divorced	16	9.2	16	9.2	
Widowed	7	4.1	10	5.8	

**Annual family income, $**					

0–20,000	14	8.2	12	7.1	χ^2^_3_ = 1.0 (.79)
20,001–40,000	34	20.0	28	16.5	
40,001–60,000	37	21.8	41	24.1	
>60,000	85	50.0	89	52.3	

**Education**					

<High school	4	2.3	7	4.0	χ^2^_4_ = 6.6 (.16)
High school	37	21.5	46	26.6	
Some college	58	33.7	39	22.5	
College degree	39	22.7	38	22.0	
Post-college degree	34	19.8	43	24.9	

**Table 2:** Physical Activity and Nutrition Variables at Baseline and 6-Month Follow-up Among Participants in a Therapeutic Lifestyle-Modification Program

**Variable**	**Intervention Group (n = 174)**				**Control Group (n = 174)**			

	**Baseline Mean (SD)**	**6-Month Follow-up Mean (SD)**	**Mean Change**	***t* a(*P* b)**	**Baseline Mean (SD)**	**6-Month Follow-up Mean (SD)**	**Mean Change**	***t* c(*P* b)**
Total steps/week	40,579 (22,631)	52,951 (24,240)	12,372	9.11 (<.0001)	43,869 (23,466)	49,530 (22,544)	5661	4.12 (<.0001)
Kcal intake/day	2092 (1030)	1534 (691)	−558	−9.45 (<.0001)	1919 (805)	1773 (777)	−146	−3.35 (.001)
Fat kcal/day, %	36.7 (6.9)	28.5 (7.0)	−8.2	−13.96 (<.0001)	34.6 (7.4)	35.6 (8.3)	1.0	1.92 (.06)
Protein kcal/day, %	15.2 (2.8)	14.4 (2.2)	−0.8	−3.74 (.0003)	14.7 (2.5)	15.4 (3.2)	0.7	2.61 (.01)
Carbohydrates kcal/day, %	48.7 (8.0)	59.2 (8.5)	10.5	15.38 (<.0001)	50.8 (8.2)	49.4 (9.6)	−1.4	−2.41 (.02)
Fruit and vegetable fiber, g/day	7.6 (4.3)	11.6 (5.6)	4.0	9.30 (<.0001)	8.3 (5.0)	8.0 (4.5)	−0.3	−1.31 (.19)
Vegetable servings/day	3.3 (2.1)	4.7 (2.6)	1.4	7.24 (<.0001)	3.4 (2.2)	3.5 (2.1)	0.1	0.44 (.66)
Fruit servings/day	1.3 (1.0)	2.2 (1.2)	0.9	10.39 (<.0001)	1.6 (1.1)	1.6 (1.1)	0	−0.39 (.69)
Whole-grain servings/day	5.4 (2.9)	6.1 (3.2)	0.7	3.11 (.002)	5.0 (2.4)	4.5 (2.3)	−0.5	−3.46 (.001)
Meat servings/day	2.1 (1.4)	1.3 (1.0)	−0.8	−8.29 (<.0001)	1.9 (1.2)	1.9 (1.1)	0	0.01 (.99)
Dietary fat, g/day	88.6 (55.3)	50.6 (33.5)	−38.0	−12.15 (<.0001)	76.8 (42.9)	71.9 (40.3)	−4.9	−2.19 (.03)
Dietary cholesterol, mg/day	216 (140)	94 (90)	−122	−14.55 (<.0001)	182 (112)	192 (140)	10	1.24 (.22)
Polyunsaturated fat, g/day	21.2 (14.0)	13.6 (8.3)	−7.6	−9.43 (<.0001)	19.3 (12.0)	17.7 (10.4)	−1.6	−2.53 (.01)
Monounsaturated fat, g/day	34.3 (21.6)	18.8 (13.1)	−15.5	−12.42 (<.0001)	29.7 (17.2)	27.9 (16.3)	−1.8	−1.99 (.048)
Saturated fat, g/day	26.3 (17.3)	13.3 (10.5)	−13.0	−13.34 (<.0001)	21.8 (12.1)	20.5 (12.0)	−1.4	−2.17 (.03)
Sodium, mg/day	2941 (1530)	2332 (1216)	−609	−7.35 (<.0001)	2712 (1233)	2486 (1135)	−226	−3.40 (.001)

a *df* = 170 for all variables except total steps/week, for which *df* = 166.

b Significance for *P* values was determined using the Bonferroni correction and set at α = .0001.

c *df* = 171 for all variables except total steps/week, for which *df* = 169.

**Table 3:** Comparison of 6-Month Change in Mean Values for Nutrition and Physical Activity Variables Among Intervention-Group (n = 174) and Control-Group (n = 174) Participants in a Therapeutic Lifestyle-Modification Program

**Variable**	**Change in Mean Values (95% Confidence Interval)**	***t_df_* (*P* a)**
Total steps/week	6711 (3026 to 10,396)	*t*_335_ = 3.81 (.0002)
Kcal intake/day	−412 (−556 to −271)	*t*_310_ = −5.69 (<.0001)
Fat kcal/day, %	−9.2 (−10.6 to −7.6)	*t*_329_ = −11.98 (<.0001)
Protein kcal/day, %	−1.5 (−2.1 to −0.8)	*t*_341_ = −4.50 (<.0001)
Carbohydrates kcal, %	11.9 (10.1 to 13.6)	*t*_328_ = 13.42 (<.0001)
Fruit and vegetable fiber, g/day	4.3 (3.3 to 5.3)	*t*_284_ = 8.59 (<.0001)
Vegetable servings/day	1.3 (0.9 to 1.9)	*t*_302_ = 5.72 (<.0001)
Fruit servings/day	0.9 (0.6 to 1.1)	*t*_337_ = 8.01 (<.0001)
Whole-grain servings/day	1.2 (0.7 to 1.7)	*t*_310_ = 4.55 (<.0001)
Meat servings/day	−0.8 (−1.0 to −0.5)	*t*_341_ = −6.23 (<.0001)
Dietary fat, g/day	−33.1 (−40.6 to −25.7)	*t*_303_ = −8.74 (<.0001)
Dietary cholesterol, mg/day	−132 (−153 to −108)	*t*_341_ = −11.35 (<.0001)
Polyunsaturated fat, g/day	−6.0 (−8.1 to −4.1)	*t*_316_ = −6.01 (<.0001)
Monounsaturated fat, g/day	−13.7 (−16.6 to −10.6)	*t*_310_ = −8.90 (<.0001)
Saturated fat, g/day	−11.6 (−13.9 to −9.3)	*t*_290_ = −10.06 (<.0001)
Sodium, mg/day	−383 (−590 to −176)	*t*_322_ = −3.69 (.0003)

a Significance for *P* values was determined using the Bonferroni correction and set at α = .0001.

**Table 4:** Health Risk Factors at Baseline and 6-Month Follow-up Among Participants in a Therapeutic Lifestyle-Modification Program

**Health Risk Factor**	**Intervention (n = 174)**				**Control Group (n = 174)**			

	**Baseline Mean (SD)**	**6-Month Follow-up Mean (SD)**	**Mean Change**	***t_df_* (*P* a)**	**Baseline Mean (SD)**	**6-Month Follow-up Mean (SD)**	**Mean Change**	***t_df_* (*P* a)**
Body mass index	33.3 (8.0)	31.7 (8.1)	−1.6	*t*_172_ = −11.53 (<.0001)	31.4 (9.0)	31.1 (9.2)	−0.3	*t*_172_ = −3.01 (.003)
Weight, kg	93.3 (24.1)	88.8 (24.0)	−4.5	*t*_172_ = −11.83 (<.0001)	87.7 (25.9)	87.1 (26.0)	−0.6	*t*_173_ = −1.64 (.10)
Body fat, %	40.6 (8.8)	38.2 (9.6)	−2.4	*t*_172_ = −8.20 (<.0001)	37.9 (10.3)	37.1 (10.5)	−0.8	*t*_172_ = −3.20 (.002)
Systolic blood pressure, mm Hg	129 (16)	124 (18)	−5	*t*_172_ = −5.60 (<.0001)	128 (17)	124 (18)	−4	*t*_171_ = −4.98 (<.0001)
Diastolic blood pressure, mm Hg	78.3 (9.2)	72.8 (9.7)	−5.5	*t*_172_ = −8.35 (<.0001)	76.7 (9.6)	72.9 (9.7)	−3.8	*t*_172_ = −6.49 (<.0001)
Resting heart rate, beats/min	73.1 (10.2)	69.6 (10.6)	−3.5	*t*_167_ = −5.30 (<.0001)	72.1 (10.6)	70.8 (10.3)	−1.3	*t*_167_ = −1.52 (.13)
Glucose, mg/dL	103 (23)	100 (20)	−3	*t*_171_ = −2.79 (.006)	100 (19)	99 (22)	−1	*t*_167_ = −0.29 (.77)
Cholesterol, mg/dL	193 (33)	199 (34)	6	*t*_171_ = 2.88 (.004)	190 (39)	201 (39)	11	*t*_167_ = 4.81 (<.0001)
High-density lipoprotein, mg/dL	45.0 (12.2)	46.4 (11.8)	1.4	*t*_171_ = 2.70 (.008)	45.0 (10.4)	47.8 (10.4)	1.8	*t*_167_ = 5.38 (<.0001)
Low-density lipoprotein, mg/dL	122 (29)	127 (29)	5	*t*_168_ = 2.83 (.005)	121 (33)	130 (34)	9	*t*_166_ = 4.12 (<.0001)
Triglycerides, mg/dL	133 (102)	128 (78)	−5	*t*_171_ = −0.91 (.37)	115 (86)	117 (69)	2	*t*_167_ = 0.31 (.76)

** **	**Baseline Median (Range)**	**6-Month Follow-up Median (Range)**	**Mean Change**	**Signed Rank S^b^(*P* a)**	**Baseline Median (Range)**	**6-Month Follow-up Median (Range)**	**Mean Change**	**Signed Rank S^b^(*P* a)**

C-reactive protein, mg/dL^b^	283.0 (2.6-1320.0)	217.5 (0.2-419.0)	−66.5	−1059.5 (.06)	228.6 (13.5-1356.8)	226.6 (0.4-1354.5)	−2.0	−814 (.17)

a Significance for *P* values was determined using the Bonferroni correction and set at α = .0001.

b C-reactive protein mean change scores violated the assumption of normality; thus, median and range scores are reported. The Wilcoxon signed rank test was used to test for differences in medians within groups.

**Table 5:** Comparison of 6-Month Change in Mean Values of Health Risk Factors Among Intervention-Group (n = 174) and Control-Group (n = 174) Participants in a Therapeutic Lifestyle-Modification Program

**Health Risk Factor**	**Change in Mean Values (95% Confidence Interval)**	***t_df_* (*P* a)**
Body mass index	−1.3 (−1.65 to −0.96)	*t*_320_ = −7.39 (<.0001)
Weight, kg	−3.9 (−5.0 to −2.8)	*t*_345_ = −6.89 (<.0001)
Body fat, %	−1.6 (−2.3 to −0.9)	*t*_321_ = −4.60 (<.0001)
Systolic blood pressure, mm Hg	−1 (−4 to 2)	*t*_342_ = −0.66 (.51)
Diastolic blood pressure, mm Hg	−1.7 (−3.5 to −0.0)	*t*_338_ = −2.03 (.04)
Resting heart rate, beats/min	−2.2 (−4.4 to −0.1)	*t*_334_ = −2.19 (.03)
Glucose, mg/dL	−2 (−6 to 0.4)	*t*_338_ = −1.72 (.09)
Cholesterol, mg/dL	−5 (−11 to 1)	*t*_338_ = −1.79 (.08)
High-density lipoprotein, mg/dL	−1.4 (−2.9 to −0.0)	*t*_338_ = −2.01 (.045)
Low-density lipoprotein, mg/dL	−4 (−9 to 2)	*t*_320_ = −1.41 (.16)
Triglycerides, mg/dL	−7 (−22 to 9)	*t*_338_ = −0.83 (.40)
C-reactive protein, mg/dL	−68.5 (−94 to 40)	*t*_326_ = −0.80 (.42)

a Significance for *P* values was determined using the Bonferroni correction and set at α = .0001.

**Table 6:** Health Risk Prevalence and Change in Mean Scores at 6-Month Follow-up Among Intervention-Group Participants (n = 174) in a Therapeutic Lifestyle-Modification Program

**Health Risk Status**	**No. (%) of Participants at Baseline**	**No. (%) of Participants at 6-Month Follow-up**	**χ^2^_1_ a (*P* b)**	**Baseline Mean Score**	**Follow-up Mean^c^ Score**	**Mean Change**	***t*_df_ (*P* b)**

**Body mass index (kg/m^2^)**							

Underweight (<18.5)	0 (0.0)	1 (0.6)	4.0 (.04)	---	---	---	---
Normal (18.5-24.9)	24 (13.8)	36 (20.7)		22.74	21.90	−0.84	*t*_24_ = −2.25 (.03)
Overweight (25.0-29.9)	45 (25.9)	47 (27.0)		27.70	26.14	−1.56	*t*_45_ = −5.68 (<.0001)
Obese (≥30.0)	105 (60.3)	90 (51.7)		38.16	36.35	−1.81	*t*_104_ = −10.21 (<.0001)

**Systolic blood pressure (mm Hg)**							

Normal (<120)	52 (29.9)	76 (43.7)	5.0 (.02)	111.12	109.51	−1.61	*t*_51_ = −1.08 (.28)
Prehypertensive (120-139)	79 (45.4)	64 (36.8)		129.22	123.88	−5.34	*t*_79_ = −3.79 (.0002)
High (140-159)	35 (20.1)	28 (16.1)		147.56	137.60	−9.96	*t*_35_ = −4.71 (<.0001)
Dangerous (≥160)	8 (4.6)	6 (3.4)		167.50	158.62	−8.88	*t*_8_ = −2.01 (.046)

**Diastolic blood pressure (mm Hg)**							

Normal (<80)	97 (55.8)	131 (75.3)	15.2 (<.0001)	71.90	68.09	−3.81	*t*_96_ = −4.75 (<.0001)
Prehypertensive (80-89)	55 (31.6)	35 (20.1)		83.31	78.58	−4.73	*t*_55_ = −4.36 (<.0001)
High (90-99)	20 (11.5)	7 (4.0)		93.25	76.80	−16.45	*t*_20_ = −9.15 (<.0001)
Dangerous (≥100)	2 (1.2)	1 (0.6)		---	---	---	---

**Total cholesterol (mg/dL)**							

Normal (<200)	105 (60.3)	94 (54.0)	1.6 (.20)	171.93	183.69	11.76	*t*_103_ = 4.58 (<.0001)
Borderline (200-239)	54 (31.0)	60 (34.5)		215.92	218.92	3.00	*t*_54_ = 0.94 (.35)
High risk (≥240)	15 (8.6)	20 (11.5)		257.67	234.93	−22.74	*t*_15_ = −3.42 (.001)

**Low-density lipoprotein (mg/dL)**							

Optimal (<100)	42 (24.1)	30 (17.2)	3.1 (.08)	85.02	103.07	18.05	*t*_39_ = 5.07 (<.0001)
Above optimal (100-129)	70 (40.2)	69 (39.7)		115.73	121.94	6.21	*t*_68_ = 2.43 (.02)
Borderline (130-159)	45 (25.9)	53 (30.5)		143.27	145.18	1.91	*t*_45_ = 0.61 (.54)
High (160-189)	14 (8.0)	17 (9.8)		172.64	151.43	−21.21	*t*_14_ = −3.81 (.0002)
Very high (≥190)	3 (1.7)	5 (2.9)		---	---	---	---

**High-density lipoprotein (mg/dL)**							

High (≥60)	19 (10.9)	20 (11.5)	3.4 (.06)	69.16	68.21	−0.95	*t*_19_ = −0.64 (.52)
Normal (40-59)	84 (48.3)	104 (59.8)		48.52	48.78	0.26	*t*_83_ = 0.24 (.81)
Low (<40)	71 (40.8)	50 (28.7)		34.33	37.87	3.54	*t*_70_ = 4.43 (<.0001)

**Triglycerides (mg/dL)**							

Normal (<150)	124 (71.3)	129 (74.1)	0.3 (.56)	88.68	100.05	11.37	*t*_123_ = 2.40 (.02)
Borderline (150-199)	25 (14.4)	22 (12.6)		171.52	152.58	−18.94	*t*_24_ = −1.65 (.10)
High (200-499)	23 (13.2)	22 (12.6)		283.22	238.30	−44.92	*t*_23_ = −4.06 (<.0001)
Very high (≥500)	2 (1.2)	1 (0.6)		---	---	---	---

**Glucose (mg/dL)**							

Normal (<110)	136 (78.2)	141 (81.0)	0.5 (.46)	93.67	92.77	−0.90	*t*_135_ = −0.78 (.44)
Impaired fasting glucose (110-125)	17 (9.8)	16 (9.2)		114.41	108.56	−5.85	*t*_16_ = −1.77 (.08)
Diabetes (≥126)	21 (12.1)	17 (9.8)		152.00	137.90	−14.10	*t*_21_ = −4.85 (<.0001)

a Mantel-Haenszel chi-square test was used to test differences within risk status categories.

b Significance for *P* values was determined using the Bonferroni correction and set at α = .0001.

c Follow-up means are from the same individuals in each baseline risk category.

**Table 7:** Health Risk Prevalence and Change in Mean Scores at 6-Month Follow-up Among Control-Group Participants (n = 174) in a Therapeutic Lifestyle-Modification Program

**Health Risk Status**	**No. (%) of Participants at Baseline**	**No. (%) of Participants at 6-Month Follow-up**	**χ^2^_1_ a (*P* b)**	**Baseline Mean Score**	**Follow-up Mean^c^ Score**	**Mean Change**	***t_df_* (*P* b)**

**Body mass index (kg/m^2^)**							

Underweight (<18.5)	0 (0.0)	1 (0.6)	0.04 (.84)	---	---	---	---
Normal (18.5-24.9)	43 (24.7)	44 (25.3)		23.15	23.04	−0.11	*t*_43_ = −0.52 (.60)
Overweight (25.0-29.9)	56 (32.2)	54 (31.0)		27.42	27.10	−0.32	*t*_55_ = −1.55 (.12)
Obese (≥30.0)	75 (43.1)	75 (43.1)		39.11	38.62	−0.49	*t*_75_ = −2.84 (.005)

**Systolic blood pressure (mm Hg)**							

Normal (<120)	60 (34.5)	81 (46.6)	4.7 (.03)	111.07	109.67	−1.40	*t*_60_ = −0.92 (.36)
Prehypertensive (120-139)	69 (39.7)	64 (36.8)		128.72	123.52	−5.20	*t*_68_ = −3.58 (.0004)
High (140-159)	40 (23.0)	21 (12.1)		149.51	141.64	−7.87	*t*_39_ = −4.16 (<.0001)
Dangerous (≥160)	5 (2.9)	8 (4.6)		165.40	157.00	−8.40	*t*_5_ = −1.59 (.11)

**Diastolic blood pressure (mm Hg)**							

Normal (<80)	102 (58.6)	127 (73.0)	9.3 (.002)	70.55	68.08	−2.47	*t*_101_ = −3.25 (.001)
Prehypertensive (80-89)	52 (29.9)	40 (23.0)		82.21	77.83	−4.38	*t*_52_ = −4.25 (<.0001)
High (90-99)	18 (10.3)	5 (2.9)		93.33	84.61	−8.72	*t*_18_ = −4.98 (<.0001)
Dangerous (≥100)	2 (1.2)	2 (1.2)		---	---	---	---

**Total cholesterol (mg/dL)**							

Normal (<200)	107 (61.5)	86 (49.4)	6.7 (.01)	164.20	183.74	19.54	*t*_102_ = 6.40 (<.0001)
Borderline (200-239)	52 (29.9)	60 (34.5)		219.77	227.90	8.13	*t*_51_ = 2.04 (0.04)
High risk (≥240)	15 (8.6)	28 (16.1)		260.47	236.67	−23.8	*t*_15_ = −3.14 (0.002)

**Low-density lipoprotein (mg/dL)**							

Optimal (<100)	48 (27.6)	34 (19.5)	6.3 (.01)	81.43	100.38	18.95	*t*_43_ = 4.22 (<.0001)
Above optimal (100-129)	58 (33.3)	52 (29.9)		113.48	130.12	16.64	*t*_57_ = 5.18 (<.0001)
Borderline (130-159)	46 (26.4)	50 (28.7)		142.67	148.11	5.44	*t*_46_ = 1.54 (.13)
High (160-189)	17 (9.8)	32 (18.4)		170.35	169.06	−1.29	*t*_16_ = −0.28 (.78)
Very high (≥190)	5 (2.9)	6 (3.4)		---	---	---	---

**High-density lipoprotein (mg/dL)**							

High (≥60)	16 (9.2)	24 (13.8)	5.2 (.02)	65.06	62.75	−2.31	*t*_16_ = −1.43 (.15)
Normal (40-59)	99 (56.9)	109 (62.6)		48.05	50.08	2.03	*t*_98_ = 3.27 (.001)
Low (<40)	59 (35.9)	41 (23.6)		33.87	39.84	5.97	*t*_54_ = 6.40 (<.0001)

**Triglycerides (mg/dL)**							

Normal (<150)	138 (79.3)	131 (75.3)	0.7 (.40)	84.02	99.76	15.74	*t*_132_ = 3.23 (.002)
Borderline (150-199)	18 (10.3)	21 (12.1)		171.50	154.72	−16.78	*t*_18_ = −1.24 (.21)
High (200-499)	17 (9.8)	21 (12.1)		259.12	208.12	−51.00	*t*_17_ = −3.71 (.0003)
Very high (≥500)	1 (0.6)	1 (0.57)		---	---	---	---

**Glucose (mg/dL)**							

Normal (<110)	150 (86.2)	152 (87.4)	0.0 (.98)	94.64	94.41	−0.23	*t*_146_ = −2.98 (.77)
Impaired fasting glucose (110-125)	17 (9.8)	13 (7.5)		112.94	115.56	2.62	*t*_16_ = 0.68 (.50)
Diabetes (≥126)	7 (4.0)	9 (5.2)		175.57	168.33	−7.24	*t*_6_ = −1.16 (.25)

a Mantel-Haenszel chi-square test was used to test differences within risk status categories.

b Significance for *P* values was determined using the Bonferroni correction and set at α = .0001.

c Follow-up means are from the same individuals in each baseline risk category.
